# Construction of an individual socioeconomic status index for analysing inequalities in colorectal cancer screening

**DOI:** 10.1371/journal.pone.0278275

**Published:** 2022-12-01

**Authors:** Mercedes Vanaclocha-Espí, Marina Pinto-Carbó, Javier Martín-Pozuelo, Paula Romeo-Cervera, Rosana Peiró-Pérez, Carmen Barona, Francisco Ortiz, Andreu Nolasco, Susana Castán, Dolores Salas, Ana Molina-Barceló

**Affiliations:** 1 Foundation for the Promotion of Health and Biomedical Research-Public Health Research FISABIO–Public Health Research, Valencia, Spain; 2 General Directorate of Public Health, Valencian Community, Spain; 3 Spanish Consortium for Research on Epidemiology and Public Health (CIBERESP), Instituto de Salud Carlos III, Madrid, Spain; 4 Health Insurance Service and the SIP of the Conselleria de Sanitat, Valencian Community, Spain; 5 University of Alicante, Alicante, Spain; Oswaldo Cruz Foundation, BRAZIL

## Abstract

**Objective:**

To construct an individual socioeconomic status index (ISESI) with information available in the Population Information System of the Region of Valencia, Spain, and use it to analyse inequalities in a colorectal cancer screening programme (CRCSP).

**Methods:**

Cross-sectional study of men and women aged between 50 and 75 at the time of the study (2020) that were selected from the target population of the Region of Valencia CRCSP. (study sample 1,150,684). First, a multiple correspondence analysis was performed to aggregate information from the Population Information System of the Region of Valencia into an ISESI. Second, data from the 2016 Region of Valencia Health Survey were used for validation, and finally the relationship between CRCSP participation and the ISESI was analysed by logistic regression models.

**Results:**

The variables included in the index were nationality, employment status, disability, healthcare coverage, risk of vulnerability and family size. The most important categories for determining the highest socioeconomic status were being employed and not being at risk of social vulnerability, and being unemployed and at risk of social vulnerability for determining the lowest socioeconomic status. Index validation demonstrated internal and external coherence for measuring socioeconomic status. The relationship between CRCSP participation and the ISESI categorised by quartile (Q) showed that Q4 (the lowest socioeconomic status) was less likely to participate OR = 0.769 (0.757–0.782) than Q1 (the highest socioeconomic status), and the opposite was found for Q2 OR = 1.368 (1.347–1.390) and Q3 OR = 1.156 (1.137–1.175).

**Conclusions:**

An ISESI was constructed and validated using Population Information System data and made it possible to evaluate inequalities in colorectal cancer screening.

## Introduction

Health equity is defined as “the absence of unfair and avoidable or remediable differences in health among population groups defined socially, economically, demographically or geographically” [1]. Analysing social determinants of health and guaranteeing the health equity perspective has become one of the main challenges for developed countries newlyand is the focus of policies in many territories [2].

Colorectal cancer (CRC) screening programmes (CRCSP) is a widely accepted public health policy in Europe. In Spain, CRCSP are population-based and aimed at men and women aged between 50 and 69, in line with the recommendations of European CRC screening guidelines [3]. The European Commission recommends analysing inequalities in CRC screening [4]. At present, most CRCSP in Spain are not subject to a systematic assessment that includes socioeconomic variables [5], principally due to the unavailability of individual socioeconomic status (SES) indicators [6].

SES is a multidimensional social determinant of health. The development of SES indicators including different socioeconomic dimensions is important in order to provide evidence-based information on social inequalities in health to support decision-making. Some previous studies in Spain have constructed multidimensional area-level deprivation indexes. The first area-level deprivation index using census data in Spain was developed by the MEDEA project and was applicable to large cities [7]. In 2011, the Spanish Society of Epidemiology replicated this methodology to create an indicator for the entire Spanish territory [8]. These indices have been used to assess and analyse socioeconomic inequalities in health at the territory level.

Some studies in Spain have analysed inequalities in CRCSP participation by using this type of area-level deprivation indexes [9], but the lack of individual SES indicators has limited interpretation of the inequalities revealed. Other studies have focused on analysing inequalities in such programmes with individual SES variables based on information collected from a population sample via surveys [10]. Although these studies are useful, data collection requires a large amount of effort and this hinders their use in periodic and systematic assessments of inequalities in cancer screening, as recommended by the European Commission [4].

The Population Information System (PIS) of the Region of Valencia, Spain, includes a Segmented, Integrated and Geographic Population Analysis code (SIGPAC) [11], which collects personalised information on variables related to healthcare coverage and socioeconomic characteristics for the entire population with the right to healthcare coverage, including country of origin, income, employment status or risk of social vulnerability. The data is updated periodically and comes from official and accurate sources, and these are some of the great advantages of this information system. Therefore, it has the potential to characterise, in socioeconomic terms, the population registered in the PIS.

As it is important to have individual indicators that measure SES in order to identify inequalities in health, the purpose of this study was to construct an individual socioeconomic status index (ISESI) based on information available in the SIGPAC. In addition, this study intends to use the ISESI to evaluate inequalities in CRCSP participation in the Region of Valencia.

## Materials and methods

### Design

Cross-sectional study to construct an individual socioeconomic status index (ISESI) based on information available in the Patient Information System (PIS) of the Region of Valencia, Spain.

### Study population

The study population (n = 1,208,515) was composed of men and women aged between 50 and 75 at the time of the study (2020) that were selected from the target population of the most recently completed round in 2020 of the Region of Valencia colorectal cancer screening programme (CRCSP).

Individuals with non-updated information in February 2020 in the PIS (n = 47,298) were excluded, as well as people with inconsistent date of birth data between the CRCSP information system and the SIGPA (n = 151) and people with unknown date in any of the variables contemplated for the creation of the ISESI (n = 10,382). The final study sample comprised 1,150,684 people.

#### Data sources

The study population was selected from the Region of Valencia CRCSP Information System. It was subsequently crossed with the Population Information System (PIS) of the Regional Ministry of Universal and Public Health of the Region of Valencia and the derived SIGPAC in 2020, thereby obtaining information on healthcare coverage and socioeconomic characteristics. The SIGPAC is updated periodically on the last day of the month including only those people who are registered in the PIS. It is a fixed photo, so the possible variation of a citizen’s data throughout the month is not reflected.

#### Study variables

Table 1 shows the variables available in the SIGPAC, in addition to an operative definition and the categories included in each of them [11].

**Table 1:** Variables available in the population information system and categorisation for ISESI construction.

Name of SIGPAC variables	Definition	SIGPAC categories	Name of ISESI variables	Recategorisation for ISESI construction
Date of birth	Date of birth	year/month/day (yyyy/mm/dd)		
Sex	Sex	Male, Female	Sex	Male, Female
Geopolitical groups	Country of origin	Classification in relation with the RV or another Spanish region and with continents (9 categories) and subregions (24 categories)	NU	NU
Migrations	Change of residence to another municipality within the RV or outside of the RV.	Non-migrant, resident immigrant from a foreign country, medium-term immigrant from a foreign country, long-term immigrant from a foreign country, recent immigrant from another Region of Spain, medium-term immigrant from another Region of Spain, long-term immigrant from another Region of Spain, any other situation	NU	NU
Nationality	Legal recognition by the Spanish State of the rights and duties of Spanish citizens	Spanish, Not Spanish, Unknown	Nationality	Spanish, Not Spanish
Residency status	Residency status by amount of time that the person has been officially registered as living in a municipality of the RV	Ordinary resident, stay in the RV, regular tourist, sporadic tourist, another situation	NU	NU
Assigned healthcare service	Organisation that includes the population receiving healthcare from a referral hospital	24 healthcare services	NU	NU
Assigned healthcare area	Level immediately below healthcare service	X healthcare areas	NU	NU
Assigned healthcare center	Level immediately below healthcare area	X healthcare center	NU	NU
Registered residence	Administratively and legally recognised residence in a municipality of a Spanish administrative territory	Registered as living in the RV, not registered as living in the RV > 1 month, not registered as living in the RV < 1 month	NU	NU
Relationship with employment status	Relationship with employment status	B. Cannot work due to age, C. Works, D. Does not work but is able to do so, E. Another situation, O. Any other situation, P. B and disabled, Q. C and disabled, R. D and disabled, S. E and disabled, T. O and disabled	Employment status	Retired (B+P), employed (C+Q), unemployed (D+R), Excluded (E, O, S, T)
			Disability	Not disabled (B+C+D), disabled (P+Q+R)
Healthcare financing and coverage	Types of health insurance according to the origin of healthcare protection and the scope of healthcare benefits	10. Spanish national or regional social security protection, 20. Protection from the RV, 30. Public mutualism, 40. European Health Insurance Card, 51. Private mutualism, 52. Private mutualism, 60. Not authorised	Healthcare coverage	Social security (10+20), public mutualism (30), private mutualism (51+52), European Health Insurance Card (40), Excluded (60)
Healthcare insurance groups and subgroups	Grouping by type of healthcare insurance	A1. International agreements, registered with social security (SS), A2. European Health Insurance Card, A3. Registered with SS and private mutualism, B1. Extension of Healthcare, B2. Low income and Spanish health insurance card, B3. Extension upon request, B4. Other authorisations from the regional Ministry, C1. Authorisation expired, C2. Not authorised, C3. Private mutualisms, C4. No income RV, C5. Undocumented foreign immigrants, OO. Unclassified	NU	NU
SIGPAC vulnerability profile	Social, economic and healthcare vulnerability status.	0. No risk, 1. Unemployed, 2. Undocumented foreign immigrants that are beneficiaries of an insured person, 3. No income, 4. Undefined (unclassifiable), 5. Undocumented foreign immigrants, 6. Unidentified persons under protection, 7. Unemployed and at objective risk	Risk of vulnerability	No risk (0), Risk due to unemployment (1+7), Risk due to low income (2+3+5), Excluded (4, 6)
Living unit	Persons living within the same codified unit	Family unit, no family unit (32 categories)	NU	NU
Family unit composition	Composition according to the number of adults and minors living together in a family unit	No family unit, one adult, one adult with N minors, two adults without minors, two adults with N minors, >2 adults without minors, > 2 adults with N minors	NU	NU
Family size	Family size according to the number of people living together in a family unit	No family unit, small (<3 persons), medium (3 to 4 persons), large (>4 persons)	Family size	No family unit, Small family size, Medium family size, Large family size
Contribution to prescription charges and annual income	% Contribution to payment of pharmacy services according to annual income	0%-income not available, 10%-income not available, 10%-income <€18,000, 40%-income <€18,000, 40%-income not available, 50%-income €18,000–100,000, 50%-income not available, 60%-income >€100,000, 60%-income >€100,000, 100%-income not available	NU	NU
Chronicity	Citizens status as regards chronic conditions	Not chronic, Level 1, Level 2, Level 3	NU	NU

SIGPAC, Integrated and Geographic Population Analysis code; RV, Region of Valencia; ISESI, Individual Socioeconomic Status Index; NU, not used for constructing the ISESI

To select the candidate SIGPAC variables to be included in the ISESI, first of all their conceptual capacity to measure individual SES was assessed on the basis of their operative definition. The following variables were excluded in this first process: residency status, the assigned healthcare service area and center. Subsequently, variables with a high number of unknown cases were ruled out. Consequently, contribution to prescription charges and annual income were discarded (more than 10% unknown). The rest of the variables included in SIGPAC were analyzed.

### Statistical analysis

#### Construction of the deprivation index

A multiple correspondence analysis (MCA) was performed to create the ISESI. MCA allows for the analysis of potential relationships between categories of more than two qualitative variables, providing a numerical representation of the relationships between the categories and identifying homogenous subgroups and influences [12].

As a result of MCA, it was decided to take into account the first three dimensions as they accumulated the highest percentage of total variability. The results of each dimension were interpreted to identify whether the categories were grouped based on socioeconomic characteristics. Finally, the first dimension was selected to create the ISESI as it explained the highest percentage of variability and had the strongest conceptual relationship with SES. The results of dimensions 2 and 3 can be seen in a supporting file [S1 Table].

The variable categories were represented in a bidimensional space corresponding to dimensions 1 and 2, and the percentage contribution of each category was shown in dimension 1.

The variables contemplated for the creation of the ISESI were: Nationality, Migration, Geopolitical groups, Healthcare coverage, Healthcare insurance groups and subgroups, Employment status, Disability, Risk of vulnerability, Family size, Living unity, Family unit composition, Sex, Age at 2020 and Chronicity. Details about these variables and the categories used can be seen in Table 1.

The quantitative ISESI was constructed by combining the coordinate values of the categories included in dimension 1. Additionally, the ISESI was categorized in four groups using quartiles independently for each sex.

Firstly, the MCA was applied to the sample stratified by sex. As the coordinates of the first dimension of the MCA model showed very little difference when the sample was separated by sex [S2 Table], the entire sample was used to construct the final model.

Finally, the ISESI was composed of 6 variables and 18 categories, resulting from the combination of variables that collected the highest percentage of viability. These are: Nationality; Healthcare coverage, Employment status, Disability, Risk of vulnerability, Family size, Sex was not used as a variable to create the index, but it was subsequently added for categorisation.

#### Internal and external validity

To confirm the validity of the constructed index, internal coherence was analysed by calculating the distribution of ISESI categories in accordance with each of the variables that make up the categorical ISESI. In addition, external validity was verified with a different population sample. This sample was made up of participants in the 2016 Region of Valencia Health Survey aged between 50 and 69 (n = 779) and with a SIGPAC at the time of the survey. The ISESI was calculated in this population and the relationship with variables related to SES characteristics available in the survey was measured: country of birth, self-declared net monthly household income, self-declared household income, self-declared household ability to make ends meet, employment status, educational level and occupational social class. Each of these variables was described as a percentage as per the categorical ISESI.

#### Study of inequalities in participation in CRCSP through ISESI

Finally, with the aim of analysing the ISESI’s capability to assess inequalities in CRC screening, its relationship with participation in the Valencia CRCSP was studied. The population studied in this analysis was the population invited in the last round of the CRCSP carried out in the region of Valencia (n = 1,107,094). Logistic regression models were applied based on the information available in the CRCSP Information System. The response variable was participation, that is to say, whether or not the screening test was carried out. The explanatory variables were the ISESI, age upon being invited to participate in the programme (<60, ≥60 years old) and type of invitation to participate in the programme (initial when the invitation was received by a person that had never participated in the programme, or successive when the invitation was received by a person that had participated in the programme on a previous occasion). The categorical ISESI was used in the model, It was fitted for the entire sample and for the sample stratified by sex. The model presented significant difference in deviances compared to the null model. Additional logistic regression models were adjusted by each of the variables used to construct the ISESI. A significance level of 0.05 was considered. All of the analyses were carried out using the statistical program R.

## Ethics considerations

This study was approved by the Research Ethics Committee of the General Directorate of Public Health and the Advanced Public Health Research Centre (No. 20180928/06). Taking into account the project design, its large sample size, the ethics committee approved carrying out the study without requesting individualized consent from each subject, following the regulations of the Declaration of Helsinki currently in effect (October 2008, Seoul). The personal data included in this study was pseudoanonymised to guarantee the confidentiality, privacy and security of the information. The project was developed in accordance with the principles of the Declaration of Helsinki and Spanish confidentiality legislation (Spanish Organic Law 3/2018, of 5 December, on Personal Data Protection and Guaranteeing Digital Rights).

## Results

Table 2 shows the distribution of the variables used to construct the ISESI and distribution by sex.

**Table 2:** Description of the variables used to create the ISESI.

Variables	Categories	n	%
Sex^a^	Male	556232	48.34
	Female	594452	51.66
Nationality	Spanish	1066381	92.67
	Not Spanish	84303	7.33
Employment status	Retired	553295	48.08
	Unemployed	205417	17.85
	Employed	391972	34.06
Disability	Not disabled	1110611	96.52
	Disabled	40073	3.48
Healthcare coverage	Social security	1098401	95.46
	Public mutualism	10045	0.87
	European Health Insurance Card	716	0.06
	Private mutualism	41522	3.61
Risk of vulnerability	No risk	993783	86.36
	Risk due to unemployment	116641	10.14
	Risk due to low income	40260	3.5
Family size	No family unit	23735	2.06
	Small family size	514082	44.68
	Medium family size	495711	43.08
	Large family size	117156	10.18

^a^Variable not included in the multiple correspondence analysis

A total of 12 dimensions were obtained in the MCA. The first three accumulated 35.24% of total variability, and the first dimension showed the highest percentage of variability at 14.31%, followed by dimension 2 at 11.26% and dimension 3 at 9.67%. (Results not shown in tables)

**Fig 1:**
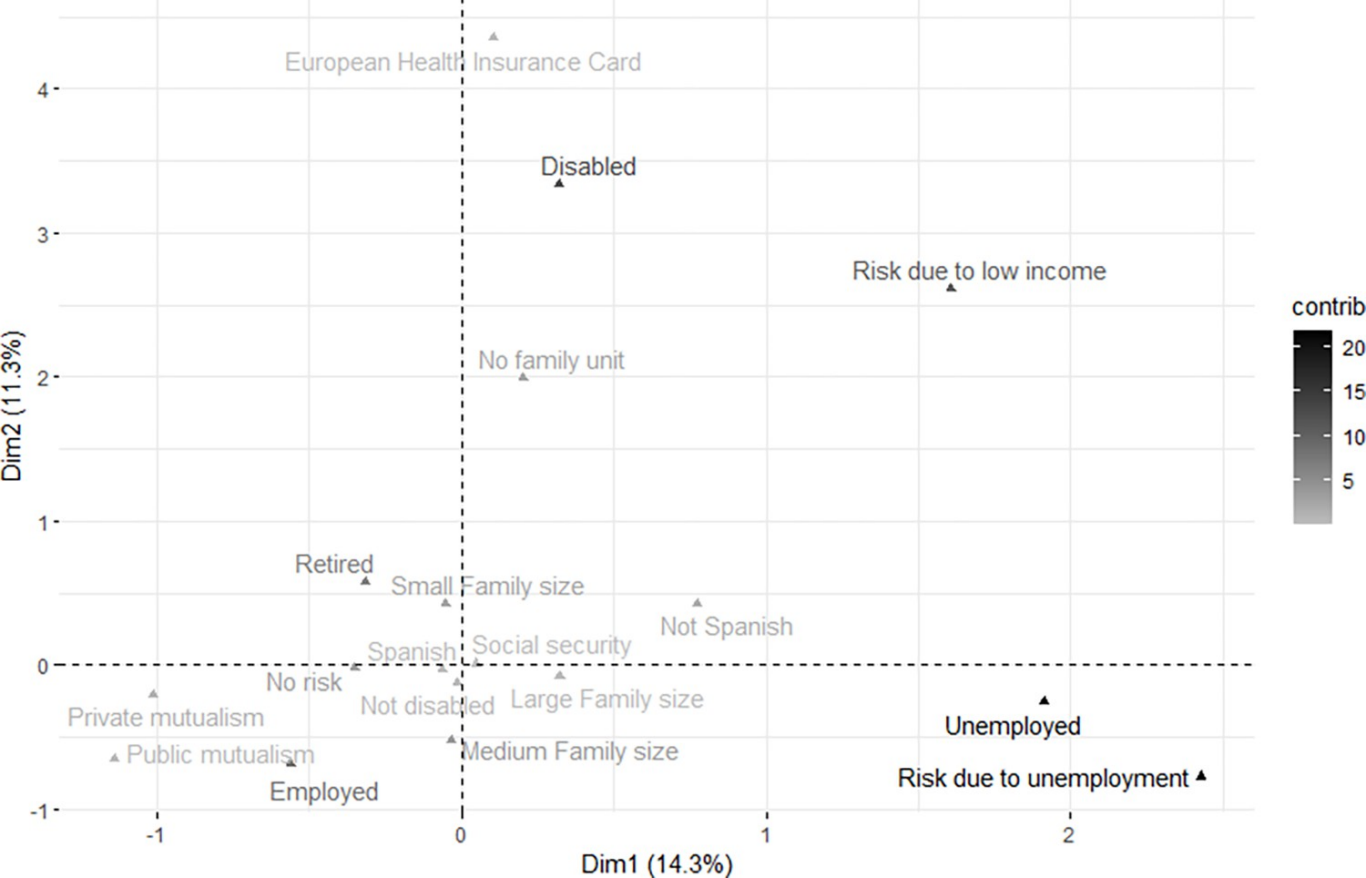
Projected cloud of variable categories and their contribution to dimension 1.

Fig 1 shows the representation of the first two dimensions obtained in the MCA and the position of the variable categories included in the study. Likewise, the contribution of each category is shown by means of colour intensity. The categories that weight the ISESI towards the highest ISESI values (retired and at risk of social vulnerability due to unemployment) are grouped between the first and fourth quadrant (right region). The categories that weight the ISESI towards the lowest ISESI values (“employed and no risk of social vulnerability”) are concentrated between the second and third quadrant (left region).

Table 3 shows the coordinates of each of the variable categories included in the first dimension, corresponding to the abscissa axis in Fig 1. In addition, the contribution of each category was included as the percentage of explained inertia. Ordered from lowest to highest, these are public mutualism healthcare coverage (-1.138), private mutualism healthcare coverage (-1.009), employed (-0.0560), no risk of social vulnerability (-0.350), retired (-0.314), Spanish nationality (-0.061), small family size (-0.052), medium family size (-0.032), no disability (-0.012), social security healthcare coverage (0.048), European Health Insurance Card (0.106), not living in a family unit (0.202), disabled (0.321), large family size (0.323), non-Spanish nationality (0.775), risk due to low income (1.606), unemployed (1.914) and risk due to unemployment (2.428). These results show that the best SES conditions have negative ISESI values.

**Table 3:** Coordinates and contribution of the variable categories included in dimension 1.

Variables	Categories	Coordinates	Contribution (%)
Nationality	Spanish	0.775	2.565
	Not Spanish	-0.061	0.203
Employment status	Retired	-0.314	2.763
	Unemployed	1.914	38.099
	Employed	-0.560	6.218
Disability	Not disabled	-0.012	0.008
	Disabled	0.321	0.210
Healthcare coverage	Social security	0.048	0.131
	Public mutualism	-1.138	0.659
	European Health Insurance Card	0.106	0.000
	Private mutualism	-1.009	2.142
Risk of vulnerability	No risk	-0.350	6.165
	Risk due to unemployment	2.428	34.816
	Risk due to low income	1.606	5.259
Family size	No family unit	0.202	0.049
	Small family size	-0.052	0.070
	Medium family size	-0.032	0.026
	Large family size	0.323	0.618

We used the dimension 1 coordinates to condense the information obtained on the involved variables to construct the ISESI. We defined the magnitude of the ISESI for each individual by adding up the coordinates of each category (Table 3). For example, the ISESI of two people, “A” and “B”, with different characteristics is shown below:

Person A: of Spanish nationality, employed, not disabled, social security healthcare coverage, no risk of vulnerability and medium family size.


$${ISESI}_{A}=-0.061-0.560-0.012+0.048-0.350-0.032=-0.967$$

Person B: of Spanish nationality, unemployed, not disabled, social security healthcare coverage, risk due to unemployment and large family size.


$${ISESI}_{B}=-0.061+1.914-0.012+0.048+2.428+0.323=4.640$$

We can see that the ISESI of person A (negative value) considers more favourable conditions than those of person B (positive value). A description of the distribution of the continuous ISESI can be seen in additional files, where an uneven distribution can be seen with a concentration of values between -1 and -0.5 [S1 Fig].

The ISESI was categorised by quartiles (Q). The internal coherence of the ISESI was assessed by analysing the distribution of the category variables that make up the index according to ISESI quartiles [S3 Table]. The results show that characteristics such as being of Spanish nationality, being employed, having social security or private mutualism coverage, not being at risk of vulnerability and having a small or medium family size are characteristic of Q1, whereas Q4 has a higher representation than other quartiles of people who are not of Spanish nationality, are unemployed, are at risk of vulnerability and have a large family size or no family unit, in addition to a lower representation of private mutualism coverage [S3 Table].

Moreover, an external validation was performed. Table 4 shows the percentage distribution by ISESI quartiles for the different categories of external variables. The trend shows that low ISESI values are associated with certain variable categories that can be identified with a high SES, and high values are associated with categories related to a low SES (from another country, low income, unemployed or in an unpaid job and difficulties making ends meet). Occupational social class and educational level had a more balanced distribution than the previously mentioned categories.

**Table 4:** Relationship of the ISESI with other SES region of Valencia health survey variables (external validity).

		ISESI categories (%)			
Variables	Categories	Q1 (highest SES)	Q2	Q3	Q4 (lowest SES)
Country of birth	Not Spanish	5.8	11.6	31.9	50.7
	Spanish	9.75	39.3	28.4	22.6
Self-declared net monthly household income	≤ €600	1.64	24.6	37.7	36.1
	€ [601, 1,200]	7.3	34.7	31.8	26.3
	€ [1,201, 1800]	10.3	41.9	21.9	25.8
	€ [1,801, 2,700]	15.8	37.6	23.8	22.8
	> €2,700	14.6	41.8	27.3	16.4
Self-declared household income	Low	4.09	28.7	29.8	37.4
	Medium-low	10.3	33.3	31.8	24.6
	Medium	11.5	40.8	26	21.7
	High-Medium High	15.8	43.9	28.1	12.3
Self-declared household ability to make ends meet	Much difficulty	3.85	23.1	32.7	40.4
	Difficulty	7.53	32.2	27.4	32.9
	Some difficulty	9.55	34.2	31.7	24.6
	Some ease	11.3	45.6	24.6	18.5
	Ease/ Very ease	14.8	14.8	27.8	15.7
Employment status	Disabled	1.72	22.4	41.4	34.5
	Unpaid	2.78	23.6	22.2	51.4
	Unemployed	3.57	11.9	9.52	75.0
	Retired	3.29	37.9	41.2	17.7
	Employed	19.2	48.2	23.1	9.45
	Other	0	36.4	36.4	27.3
Educational Level	Less Primary education	2.44	30.5	37.8	29.3
	Primary education	6.84	38.4	31.6	23.1
	Secondary education	9.09	36.7	26.1	28.0
	Higher education	20.8	36.8	21.6	20.8
Occupational social class	III	6.68	36.1	30.7	26.5
	II	11.2	49.6	21.6	17.6
	I	16.4	31.5	25.3	26.7

SES, Socioeconomic Status; ISESI, Individual Socioeconomic Status Index; Q, quartile, Occupational social class (III, Manual workers; II, Intermediate occupations and self-employed workers; I, Directors and managers and university professionals)

Table 5 shows the relationship between the ISESI and CRCSP participation. The model containing the categorical ISESI shows that women in Q2 and Q3 (OR = 1.329 and OR = 1.070, respectively) are more likely to participate than women in Q1 (the highest SES), and that women in Q4 (the lowest SES) are the least likely to participate (OR = 0.853). The same is true for men. Q2 (OR = 1.535) and Q3 (OR = 1.138) are more likely to participate than the population with a Q1 ISESI, and Q4 (OR = 0.659) is less likely to participate than the Q1 group. An additional table shows the relationship of participation with the ISESI and separately with each of the variables that comprise the ISESI. For the whole sample, the results show that the ISESI presented a better fit with participation (AIC = 966158) than all other variables, followed by employment status (AIC = 969304) [S4 Table].

**Table 5:** The ISESI and its relationship with CRCSP participation for the whole sample and by sex.

		Whole sample^a^	Female^b^	Male^b^
		OR (CI 95%)	OR (CI 95%)	OR (CI 95%)
Categorical ISESI	Q1 (highest SES)	Ref	Ref	Ref
	Q2	1.368 (1.347–1.390)	1.329 (1.300–1.358)	1.535 (1.498–1.572)
	Q3	1.156 (1.137–1.175)	1.070 (1.045–1.096)	1.138 (1.111–1.166)
	Q4 (lowest SES)	0.769 (0.757–0.782)	0.853 (0.834–0.873)	0.659 (0.644–0.675)

SES, Socioeconomic Status; ISESI, Individual Socioeconomic Status Index; Q, Quartile; OR, Odds Ratio; CI, Confidence Interval.

^a^ Adjusted for age, sex and type of invitation to participate in the programme

^b^ Adjusted for age and and type of invitation to participate in the programme

## Discussion

In this study, an individual socioeconomic status index (ISESI) was built and validated for a population aged between 50 and 69 based on information available in the Patient Information System (PIS) of the Region of Valencia, Spain. The ISESI made it possible to analyse inequalities in Valencia colorectal cancer screening programme (CRCSP) participation.

A multivariate methodology was used to give a weight to the variable categories that make up the ISESI, representing the statistical relationships between these categories. This methodology made it possible to reduce and combine the wide range of socioeconomic variables that were available in the PIS, including nationality, employment status, disability status, type of healthcare coverage, risk of vulnerability and family size. As a result, a qualitative and quantitative index was built in order to allow and facilitate the analysis of health inequalities.

It should be noted that the coefficient of variability explained in the ISESI is low, as shown in the results. This could be due to the fact that the type of information available in the PIS has the purpose of establishing the healthcare coverage rights, type of healthcare coverage and contribution to prescription charges of people registered as living in the region of Valencia. Therefore, as these rights are greatly dependent on employment status and family income, the information available in the PIS and, therefore, the information used to develop the ISESI, is focused on these characteristics. However, despite this, the percentage obtained is greater than the expected variance if random data were used, and therefore they give the ISESI validity and representativity.

The ISESI variables with the highest weight were employment status and risk of vulnerability, followed by nationality and healthcare coverage and, lastly, disability and family size. This indicates that the ISESI created in this study mainly characterises the population in accordance with their employment status and how this affects their social, economic, and healthcare vulnerability. Specifically, the nationality variable indicates the possession or absence of Spanish nationality, whose possession recognizes its relationship with the Spanish state and the recognition of a series of rights and duties of citizens in relation to health care. This variable lacks the variability associated with the country of origin, but it should be noted that the ISESI includes other variables that include the situation of social exclusion, such as Vulnerability Risk and health coverage status in the Healthcare coverage variable.

By comparing the ISESI with other SES variables and performing external validation with variables such as personal income or household income, in addition to employment status, we confirmed that the ISESI characterises the population according to socioeconomic characteristics, based on their employment and income status. In contrast, it does not appear to be related with variables traditionally used to measure SES, such as educational level or occupational social class [13]. As commented above, this is due to the type of information available in the PIS. Nonetheless, it should be noted that despite this limitation, one of the most significant advantages of the PIS is that the information is systematically collected and updated on a regular basis and coded in a uniform manner. Therefore, it is an official, stable and publicly funded information system that undergoes regular quality control [11].

The continuous ISESI has shown an uneven distribution with a higher concentration of cases in certain values, these results are consistent with the distribution of the personal characteristics included in the ISESI, which show an uneven distribution. That is why the results suggest the non-use of the ISESI in a linear way and therefore the categorization of the ISESI.The multidimensional character of the concept of SES and the growing importance of assessing health inequalities has led to the creation of SES indices using information available in various sources. There are several initiatives related to the construction of socioeconomic status indices to measure health inequalities at both the national and international level [7, 8, 14–16]. In Spain, the greatest success has been seen in the development of area-level indices based on housing census data [7, 17]. One of the most commonly used indices in the Spanish context was constructed from socioeconomic indicators at the census-section level, specifically with information on occupation type and indicators related to employment status, resulting in an ecological index [7]. These indices have at times been used to analyse the impact of area-level inequalities in cancer screening, using them from an individualised perspective [18, 19]. The ISESI created by this study has great potential as it is an individual SES index, which complements the use of ecological indices.

One limitation is that SIGPAC is automatically updated on the last day of each month, showing a fixed photo at a specific time. Although it should be noted that the PIS is population-based, and the ISESI is available for the entire population of the Region of Valencia registered in the PIS, that is, the entire resident population or that that has come into contact with the health system of the region. Our results show that combining different socioeconomic characteristics in an index to measure inequalities in CRCSP is better than using each of the population’s socioeconomic characteristics independently. These results are in line with other studies that combine several socioeconomic characteristics in a single individual index to analyse health inequalities in the adult population [20].

Some authors state that the type of socioeconomic indicator and its influence on health seems to have a different effect depending on the health problem under analysis [21–24]. One specific study shows that socioeconomic status measured in terms of income has the most significant effect on all health indicators in old age [21]. Another study shows that educational level creates inequalities in all-cause mortality, while socioeconomic variables affect cardiovascular illnesses and cancer [23]. A study performed in the UK found that the most deprived neighbourhoods presented worse conditions in terms of waiting time, repeat hospitalisation and dying in hospital than the least deprived neighbourhoods [24].

This index has been created to assess inequalities in CRC screening, among other uses. Consequently, it was developed with information on a population group aged between 50 and 69, considering the age of the target population of these programmes. The index can be incorporated to analyse inequalities in CRCSP result indicators or can be used as an SES adjustment variable. Nonetheless, the same methodology could be replicated to create indices adapted to the target populations of other public health programmes in the region of Valencia, such as the early detection of breast and cervical cancer programmes, or programmes for sexual and reproductive health, active ageing or gender violence prevention.

An initial approach to using this ISESI to identify inequalities in CRC screening has demonstrated that the population situated in Q1, i.e., with the best socioeconomic conditions, and in Q4, i.e., with the worst socioeconomic conditions, were less likely to participate than those in intermediate quartiles (Q2 and Q3). These results are in line with other studies performed in Spain [10, 18, 19, 25]. Specifically, Buron (2017) found that inequalities in CRC screening uptake in Catalonia seem to be concentrated primarily in the most disadvantaged groups, followed by the least disadvantaged ones [17]. Studies performed in the context of European screening programmes showed a participation gradient with the lowest percentages seen in the most disadvantaged social strata in the case of both men and women [26–28].

Some of the variables used to construct the ISESI, such as employment status, were used to identify inequalities in European CRCSP participation [29–31]. The results of these studies are inconsistent, as some conclude that there is no relationship between employment status and participation [29] while others do find such a relationship [31], with a trend towards lower participation in employed people compared to unemployed or retired people. In our study, we saw this trend in retired people but not in unemployed people. In addition, several studies associate income level—a variable that showed a strong correlation with the ISESI in external validation—with inequalities in participation [32–34]. They also show that the probability of participation falls as income level decreases, in line with the results of our analyses. Finally, educational level—a variable that was not used to create this index due to unavailability—has been positively linked to CRCSP participation [35, 36].

Analysing social inequalities in CRCSP participation is a complex phenomenon that requires the use of multiple and varied socioeconomic indicators in order to study these inequalities in more detail. The resulting ISESI and its inclusion in the Valencia CRCSP information system could help provide a better understanding of inequalities in CRC screening.

## Conclusions

If the ISESI created in this study were incorporated into the Valencia CRCSP Population Information System, it would be possible to systematically assess social inequalities in the impact of these programmes. This will ensure that the European Commission’s recommendations [4] are met as regards the identification of inequalities in cancer screening, thereby contributing to the design of evidence-based policies from an equity perspective. Furthermore, this methodology could be replicated in other public health programmes to favour the assessment of health inequalities, thereby making them more visible and reducing them in order to promote health equity.

## Supporting information

S1 Table Coordinates of the variable categories included in dimensions 2 and 3 in the MCA. (DOCX)

S2 Table Coordinates of the variable categories included in dimension 1 in the MCA applied by sex. (DOCX)

S3 Table Internal coherence of the variables that make up the ISESI, according to the ISESI categories. SES; Socioeconomic Status; ISESI: Individual Socioeconomic Status Index; Q: Quartile.
(DOCX)

S4 Table Logistic regression models for CRCSP participation by ISESI and variables used to create the ISESI. SES; Socioeconomic Status; ISESI: Individual Socioeconomic Status Index; Q: Quartile; OR: Odds Ratio; CI: Confidence Interval; AIC: Akaike information criterion. All models adjusted for age, sex and and type of invitation to participate in the programme.
(DOCX)

S1 Fig Continuous ISESI density graphic. (DOCX)
