# Supplementary material for: Construction of an individual socioeconomic status index for analysing inequalities in colorectal cancer screening
Source: PLoS One. 2022 Dec 1;17(12):e0278275. doi: 10.1371/journal.pone.0278275 (PMC9714724; doi:10.1371/journal.pone.0278275)
Supplement: S1 Table — (DOCX) [file pone.0278275.s001.docx]

**S1 Table. Coordinates of the variable categories included in dimensions 2 and 3 in the MCA.**

| Variables | Categories | Coordinates Dimension 2 | Coordinates Dimension 3 |
| --- | --- | --- | --- |
| Nationality | Spanish | 0.428 | -1.584 |
|  | Not Spanish | -0.034 | 0.125 |
| Employment status | Retired | 0.583 | -0.333 |
|  | Unemployed | -0.256 | 0.215 |
|  | Employed | -0.688 | 0.358 |
| Disability | Not disabled | -0.120 | -0.074 |
|  | Disabled | 3.335 | 2.046 |
| Healthcare coverage | Social security | 0.011 | -0.075 |
|  | Public mutualism | -0.650 | 0.854 |
|  | European Health Insurance Card | 4.351 | -10.233 |
|  | Private mutualism | -0.209 | 1.956 |
| Risk of vulnerability | No risk | -0.015 | -0.058 |
|  | Risk due to unemployment | -0.772 | -0.056 |
|  | Risk due to low income | 2.617 | 1.586 |
| Family size | No family unit | 1.995 | 2.667 |
|  | Small family size | 0.429 | -0.554 |
|  | Medium family size | -0.522 | 0.505 |
|  | Large family size | -0.077 | -0.247 |
