# Supplementary material for: Construction of an individual socioeconomic status index for analysing inequalities in colorectal cancer screening
Source: PLoS One. 2022 Dec 1;17(12):e0278275. doi: 10.1371/journal.pone.0278275 (PMC9714724; doi:10.1371/journal.pone.0278275)
Supplement: S2 Table — (DOCX) [file pone.0278275.s002.docx]

**S2 Table. Coordinates of the variable categories included in dimension 1 in the MCA applied by sex.**

| Variables | Categories | Coordinates Male | Coordinates Female |
| --- | --- | --- | --- |
| Nationality | Spanish | 0.942 | 0.561 |
|  | Not Spanish | -0.075 | -0.044 |
| Employment status | Retired | -0.276 | -0.363 |
|  | Unemployed | 2.397 | 1.614 |
|  | Employed | -0.466 | -0.627 |
| Disability | Not disabled | -0.008 | -0.014 |
|  | Disabled | 0.217 | 0.404 |
| Healthcare coverage | Social security | 0.038 | 0.059 |
|  | Public mutualism | -0.903 | -1.326 |
|  | European Health Insurance Card | 0.468 | -0.483 |
|  | Private mutualism | -0.854 | -1.137 |
| Risk of vulnerability | No risk | -0.332 | -0.364 |
|  | Risk due to unemployment | 2.686 | 2.221 |
|  | Risk due to low income | 2.009 | 1.342 |
| Family size | No family unit | 0.438 | -0.060 |
|  | Small family size | 0.081 | -0.195 |
|  | Medium family size | -0.162 | 0.131 |
|  | Large family size | 0.259 | 0.401 |
