# Supplementary material for: Construction of an individual socioeconomic status index for analysing inequalities in colorectal cancer screening
Source: PLoS One. 2022 Dec 1;17(12):e0278275. doi: 10.1371/journal.pone.0278275 (PMC9714724; doi:10.1371/journal.pone.0278275)
Supplement: S3 Table — SES; Socioeconomic Status; ISESI: Individual Socioeconomic Status Index; Q: Quartile. (DOCX) [file pone.0278275.s003.docx]

**S4 Table. Internal coherence of the variables that make up the ISESI, according to the ISESI categories.**

|  |  | ISESI categories | | | |
| --- | --- | --- | --- | --- | --- |
| Variables | Categories | Q1 (highest SES) | Q2 | Q3 | Q4 (lowest SES) |
| Nationality | Spanish | 100.0 | 100.0 | 87.0 | 79.6 |
|  | Not Spanish | 0.0 | 0.0 | 13.0 | 20.4 |
| Employment status | Retired | 6.0 | 99.6 | 75.1 | 24.2 |
|  | Unemployed | 0.0 | 0.0 | 1.2 | 71.4 |
|  | Employed | 94.0 | 0.4 | 23.7 | 4.4 |
| Disability | Not disabled | 99.7 | 100.0 | 90.2 | 93.3 |
|  | Disabled | 0.3 | 0.0 | 9.8 | 6.7 |
| Healthcare coverage | Social security | 86.1 | 100.0 | 98.6 | 99.8 |
|  | Public mutualism | 2.8 | 0.0 | 0.1 | 0.0 |
|  | European Health Insurance Card | 0.0 | 0.0 | 0.2 | 0.1 |
|  | Private mutualism | 11.1 | 0.0 | 1.1 | 0.1 |
| Risk of vulnerability | No risk | 100 | 100 | 98.1 | 46.2 |
|  | Risk due to unemployment | 0.0 | 0.0 | 0.0 | 41.0 |
|  | Risk due to low income | 0.0 | 0.0 | 1.9 | 12.8 |
| Family size | No family unit | 1.2 | 0.4 | 4.7 | 3.1 |
|  | Small family size | 38.8 | 76.1 | 17.6 | 36.8 |
|  | Medium family size | 59 | 23.6 | 49.5 | 40.2 |
|  | Large family size | 1.0 | 0.0 | 28.3 | 19.8 |

SES; Socioeconomic Status; ISESI: Individual Socioeconomic Status Index; Q: Quartile
