# Supplementary material for: Construction of an individual socioeconomic status index for analysing inequalities in colorectal cancer screening
Source: PLoS One. 2022 Dec 1;17(12):e0278275. doi: 10.1371/journal.pone.0278275 (PMC9714724; doi:10.1371/journal.pone.0278275)
Supplement: S4 Table — SES; Socioeconomic Status; ISESI: Individual Socioeconomic Status Index; Q: Quartile; OR: Odds Ratio; CI: Confidence Interval; AIC: Akaike information criterion. All models adjusted for age, sex and and type of invitation to participate in the programme. (DOCX) [file pone.0278275.s004.docx]

**S5 Table. Logistic regression models for CRCSP participation by ISESI and variables used to create the ISESI.**

|  |  | OR (CI 95%) | AIC | p-value |
| --- | --- | --- | --- | --- |
| ISESI | Q1 (highest SES) |  |  |  |
|  | Q2 | 1,368 (1,347-1,390) |  |  |
|  | Q3 | 1,156 (1,137-1,175) |  |  |
|  | Q4 (lowest SES) | 0,769 (0,757-0,782) | 966266 | <0,001 |
| Nationality | Not Spanish | Ref |  |  |
|  | Spanish | 1,893 (1,856-1,931) | 976158 | <0,001 |
| Employment status | Employed | Ref |  |  |
|  | Unemployed | 0.643 (0.634-0.652) |  |  |
|  | Retired | 1.049 (1.032-1.067) | 969042 | <0,001 |
| Disability | Not disabled | Ref |  |  |
|  | Disabled | 0.626 (0.608-0.645) | 979304 | <0,001 |
| Healthcare coverage | Social security | Ref |  |  |
|  | Public mutualism | 0.514 (0.484-0.545) |  |  |
|  | European Health Insurance Card | 0.636 (0.603-0.671) |  |  |
|  | Private mutualism | 0.278 (0.225-0.341) | 973638 | <0,001 |
| Risk of vulnerability | No risk | Ref |  |  |
|  | Risk due to unemployment | 0.718 (0.706-0.730) |  |  |
|  | Risk due to low income | 0.464 (0.450-0.477) | 975306 | <0,001 |
| Family size | No family unit |  |  |  |
|  | Small family size | 0.469 (0.451-0.489) |  |  |
|  | Medium family size | 1.294 (1.280-1.309) |  |  |
|  | Large family size | 0.898 (0.882-0.914) | 975350 | <0,001 |

SES; Socioeconomic Status; ISESI: Individual Socioeconomic Status Index; Q: Quartile; OR: Odds Ratio; CI: Confidence Interval; AIC: Akaike information criterion

All models adjusted for age, sex and and type of invitation to participate in the programme
