# Supplementary figures and images for: Construction of an individual socioeconomic status index for analysing inequalities in colorectal cancer screening
Source: PLoS One. 2022 Dec 1;17(12):e0278275. doi: 10.1371/journal.pone.0278275 (PMC9714724; doi:10.1371/journal.pone.0278275)

**S3 Fig. Continuous ISESI Density graphic.**


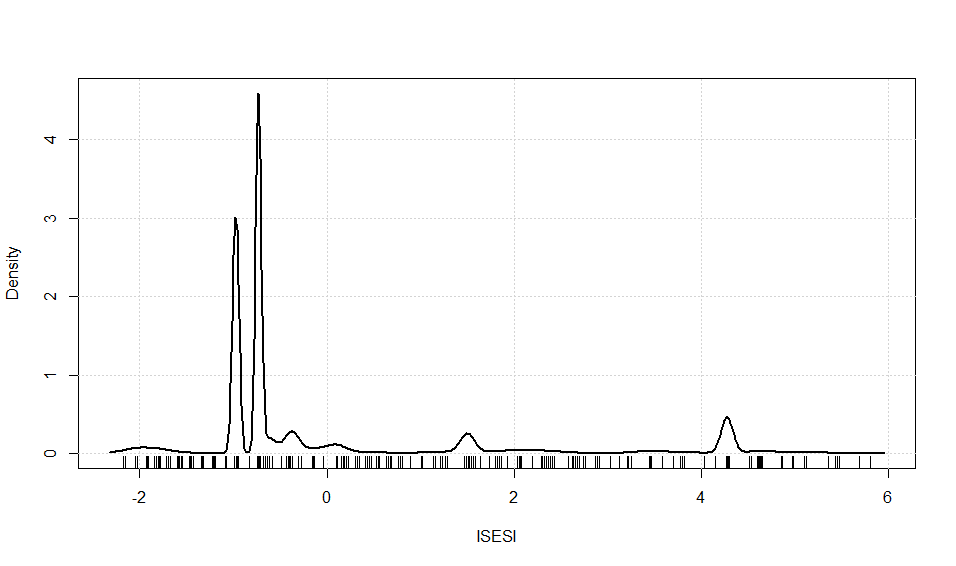

Supplement: S1 Fig — (DOCX) [file pone.0278275.s005.docx]
